# Pathologic nodal metastasis assessment using tumour‐derived molecular features in patients with lung adenocarcinoma

**DOI:** 10.1002/ctm2.1638

**Published:** 2024-04-01

**Authors:** Shijie Wang, Zhiwei Zhou, Song Wang, Rongyun Guo, Zeming Ma, Dachuan Zhao, Liang Wang, Yinan Liu, Yuanyuan Ma, Jianzhi Zhang, Sha Wang, Yedan Chen, Qiuxiang Ou, Jinfeng Chen

**Affiliations:** ^1^ Department of Thoracic Surgery II Key Laboratory of Carcinogenesis and Translational Research (Ministry of Education/Beijing) Peking University Cancer Hospital & Institute Beijing China; ^2^ Geneseeq Research Institute Nanjing Geneseeq Technology Inc. Nanjing China

Dear Editor,

Through whole‐exome sequencing (WES), we identified genomic features that correlated with pathologic lymph node metastasis (pN) and poor patient prognosis in baseline tumour tissues from lung adenocarcinoma (LUAD) patients. Our study offers valuable insights into genomic traits with increased risk of lymph node metastasis that might be utilized for a more accurate pN assessment thereby optimizing treatment decision‐making.

Tumour size and nodal staging status constitute the two fundamental elements of the tumour‐node‐metastasis (TNM) staging system. Clinical assessment of mediastinal and hilar lymph node involvement primarily leans on standard imaging modalities, including computed tomography (CT), magnetic resonance imaging (MRI), and positron emission tomography‐computed tomography (PET‐CT).^1^ Meanwhile, invasive staging approaches, such as endobronchial ultrasound transbronchial needle aspiration (EBUS‐TBNA), have demonstrated superior diagnostic effectiveness than PET‐CT.^2^ However, considerable differences in the assessment of metastatic lymph nodes between preoperative and postoperative samples were found.^3^, ^4^ Besides, while larger tumours are generally considered more closely associated with nodal metastasis in solid tumours, recent studies have shown that small adenocarcinoma tumours tend to present with lymph node metastasis.^5^, ^6^ Given the unknown risk of lymph node metastasis even with small tumours, accurate pathologic determination of nodal metastasis is of great clinical importance for optimal treatment decision‐making, for example, deciding the appropriate extent of lymphadenectomy for early‐stage non‐small cell lung cancer (NSCLC) and whether adjuvant therapy should be applied.

In this study, tumour specimens were obtained from 72 untreated LUAD patients in the curative‐intent surgery and subjected to a thorough pathologic determination of nodal status. Patients were categorized based on their pN status into two subgroups: pN‐negative (*N* = 31) and pN‐positive (*N* = 41). Among all patients, 56.9% were male and the median diagnosis age was 61 years old (range: 35–84) (Table 1). Notably, pN‐negative was more likely associated with males (*p *= .05), while pN‐positive patients were more frequently diagnosed with vessel carcinoma embolus (*p *< .001). Through WES, we delineated the mutational profile of each patient, with *TP53* (54.8% and 53.7%, respectively) being the most frequently mutated gene in the study cohort followed by epidermal growth factor receptor (*EGFR*, 35.5% and 48.8%, respectively) (Figure 1A,B). Gene‐ and pathway‐level enrichment analysis showed that Kelch‐like ECH‐associated protein 1 (*KEAP1*) and Protein Tyrosine Phosphatase Receptor Type B (*PTPRB*) mutations (*p *= .03 and *p *= .01, respectively), as well as aberrations in the NRF2 signalling pathway (*p *= .01), were significantly more enriched in pN‐negative patients (Figure 1C). The aberrant KEAP1‐NRF2 signalling has been found in various cancer types, most frequently involved in non‐small cell lung cancer.^7^ Accumulating evidence suggests that loss of *KEAP1* expression and the subsequent upregulation of NRF2 signalling may contribute to cancer progression and resistance to therapies, including chemotherapies, targeted therapies, and immune checkpoint blockade.^8^ In our study, 71.4% (5/7) of *KEAP1* mutations identified in pN‐negative patients were missense mutations with uncertain significance, and only one frameshift deletion was classified as “likely oncogenic” according to OncoKB (Table S1). Meanwhile, although pN‐positive patients were more likely to harbour genomic alterations within the cell cycle pathway and WNT pathway, the observed difference was not statistically significant. It is noteworthy that, despite a higher tumour mutation burden (TMB) observed in pN‐negative patients compared to those with positive nodal metastasis, both groups exhibited low TMB scores (median: 3.85 vs. 1.83 muts/Mb, *p *= .05) (Figure 1D). Additionally, no notable difference was observed in the chromosomal instability score (CIS) between the two subgroups of patients (median: 0.14 vs. 0.32, *p *= .14) (Figure 1E).

**TABLE 1:** Clinical characteristics of the study cohort.

Characteristic	All (*N* = 72)	pN‐negative (*N* = 31)	pN‐positive (*N* = 41)	*p*‐value
Sex				.05
Female	31 (43.1%)	9 (29.0%)	22 (53.7%)	
Male	41 (56.9%)	22 (71.0%)	19 (46.3%)	
Age at diagnosis, years				.09
median (range)	61 (35–84)	65 (43–84)	59 (35–75)	
< 60	30 (41.7%)	9 (29.0%)	21 (51.2%)	
≥60	42 (58.3%)	22 (71.0%)	20 (48.8%)	
pN stage				<.001
N0	31 (43.1%)	31 (100%)	0 (0.00%)	
N1	2 (2.78%)	0 (0.00%)	2 (4.88%)	
N2	38 (52.8%)	0 (0.00%)	38 (92.7%)	
N3	1 (1.39%)	0 (0.00%)	1 (2.44%)	
Clinical T stage				<.001
T1‐2	51 (70.8%)	10 (32.3%)	41 (100%)	
T3‐4	21 (29.2%)	21 (67.7%)	0 (0.00%)	
Smoking history				.06
Ever	32 (44.4%)	18 (58.1%)	14 (34.1%)	
Never	40 (55.6%)	13 (41.9%)	27 (65.9%)	
Tumor location				.33
LLL	9 (12.5%)	4 (12.9%)	5 (12.2%)	
LUL	20 (27.8%)	10 (32.3%)	10 (24.4%)	
RLL	16 (22.2%)	6 (19.4%)	10 (24.4%)	
RML	4 (5.56%)	0 (0.00%)	4 (9.76%)	
RUL	21 (29.2%)	9 (29.0%)	12 (29.3%)	
RUL+RLL	2 (2.78%)	2 (6.45%)	0 (0.00%)	
Vessel carcinoma embolus			<0.001	
No	48 (66.7%)	30 (96.8%)	18 (43.9%)	
Yes	24 (33.3%)	1 (3.23%)	23 (56.1%)	
Visceral pleural invasion				.10
No	39 (54.2%)	13 (41.9%)	26 (63.4%)	
Yes	33 (45.8%)	18 (58.1%)	15 (36.6%)	

Abbreviations: LLL, left lower lobe; LUL, left upper lobe; pN, pathologic lymph node metastasis; RLL, right lower lobe; RML, right middle lobe; RUL, right upper lobe.

**FIGURE 1:**
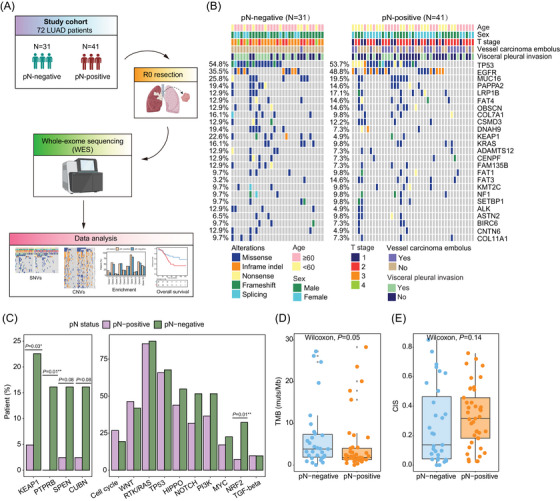
Mutational landscape of patients stratified by pathologic lymph node status. (A) An overview of the study design. Primary tumor tissues from 72 lung adenocarcinoma (LUAD) patients (the study cohort) with or without pathologic lymph node metastasis (pN) were analyzed by whole‐exome sequencing (WES) and subsequent data analysis. (B) The heatmap shows the single‐nucleotide variants (SNVs) in 72 LUAD patients categorized based on their pN status. (C) The bar plot shows the proportion of patients harbouring the specific gene/pathway alterations within the two patient subgroups in strata of pN status. (D) The box plot of tumour mutation burden (TMB) distribution between pN‐negative and pN‐positive patients. (E) The box plot of chromosome instability score (CIS) distribution between two patient subgroups.

Next, we conducted an integrative copy‐number variation (CNV) analysis on both the arm and focal gene levels. In brief, arm‐level CNVs were called if at least 60% of segments exhibited a consistent degree of copy number alteration, and focal‐level CNVs were determined based on the total copy number adjusted by the overall sample‐wide estimated ploidy (Supporting Information). Notably, gains of chromosomes 7p and 7q were more prevalent in pN‐positive than pN‐negative individuals (*p *= .008 and *p *= .04, respectively) (Figure 2A; Figure S1A). Additionally, while gains on chromosomes 16p, 16q, and 5p were also more frequently observed in pN‐positive patients, losses on chromosome 19p were more commonly identified in pN‐negative patients (Figure 2A). However, none of these arm‐level CNVs exhibited a discernible proportional difference of affected individuals between the two subgroups. Subsequently, we employed GISTIC2.0 analysis (Supporting Information) and enrichment analysis to identify likely significant focal‐level CNVs in subgroup patients. Notably, among all CNV events unveiled through WES and GISTIC analysis, only *EGFR* amplification was concurrently identified by both approaches as a top candidate implicated in nodal metastasis (Figure 2B, C). The prevalence of *EGFR* amplification exhibited a marked increase in pN‐positive compared to pN‐negative patients (61.0% vs. 25.8%, *p *= .004) (Figure 2C; Figure S1B). A strong correlation between the presence of chromosome 7p and *EGFR* amplifications was observed (Figure S1C). Collectively, our findings demonstrate the presence of diverse copy number alterations in pN‐positive patients with LUAD, including but not limited to chromosome 7 amplifications. This was consistent with our prior observation that nodal‐positive patients had higher overall chromosomal instability than nodal‐negative patients (Figure 1E).

**FIGURE 2:**
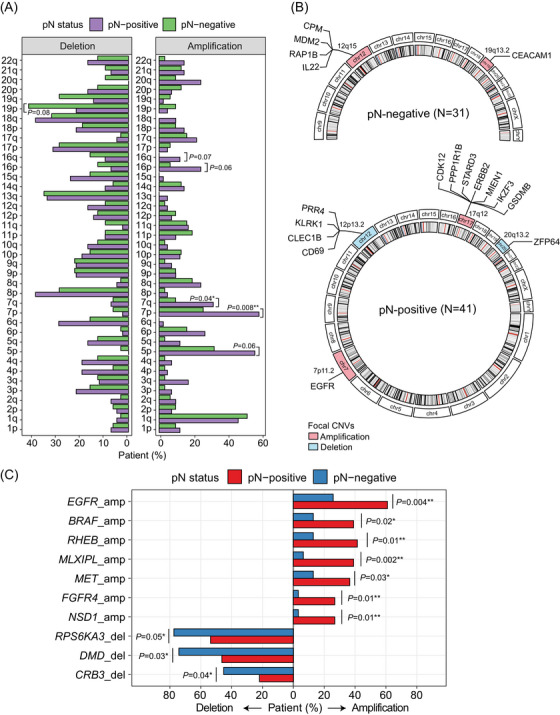
Copy‐number variant analysis in patients categorized based on pathologic lymph node status. (A) The bar plot shows the proportion of patients categorized by their pathologic lymph node metastasis (pN) status harbouring specific arm‐level copy‐number variants (CNVs). (B) The enrichment of arm‐level CNV and the corresponding focal gene‐level genomic alterations in pN‐negative and pN‐positive patients was calculated by the GISTIC2.0 algorithm. (C) The bar plot shows the proportion of patients with specific focal gene‐level CNVs.

Leveraging the comprehensive mutational landscape revealed through WES, we subsequently assessed the patient's clinical/molecular features, with an aim to identify molecular phenotypes that could enhance patient prognostic stratification. Individuals with positive pN status demonstrated a reduced overall survival (OS) compared to their pN‐negative counterparts, although this difference did not attain statistical significance likely attributed to the small cohort size (*p *= .08) (Figure S2A). However, it was worth noting that patients with small tumours (T1‐2) exhibited a less favourable OS compared to those with large tumours (T3‐4), and the observed trend was unlikely to be affected by pN status (Figure S2B,C). Conversely, pN‐negative patients present a consistent trend toward prolonged survival than pN‐positive patients, irrespective of tumour size (Figure S2A,D). Collectively, these results, which take into account the intricate interplay between pN status and tumour size, suggest that pN status likely exerts a greater influence on the overall survival probability of patients than tumour size. For molecular features, only chromosome 7p amplification (X7p_amp) enriched in pN‐positive patients was identified as a prognostic biomarker significantly associated with OS at univariate analysis (hazard ratio [HR]: 2.1; 95% confidence interval [CI]: 1−4.2; *p *= .04) (Table S2). X7p‐amplified patients had considerably shorter median OS than those without the alteration (Figure 3A), and a comparable pattern was noted among patients stratified by focal *EGFR* amplification status (Figure 3B). However, none of the other previously identified clinical/molecular features that tended to enrich in either one of the subgroups were significantly associated with prognosis (Figure S2E–I). These observations suggest that gains on chromosome 7p, particularly *EGFR* amplification, were likely driver events for nodal metastasis and closely correlated with an unfavourable prognosis in these patients. On the other hand, several arm‐level CNVs, including gains on chromosome 7q, 5p, 16p and 16q, as well as loss on chromosome 19p, exhibited a discernible difference between nodal‐negative and nodal‐positive patients, though the statistical power of our analysis might be affected by the limited cohort size. Further validation using a more extensive cohort is necessary to evaluate the correlation between these arm‐level CNVs, pN status, and patient prognosis. Notably, the impact of single‐nucleotide variations (SNVs) on driving nodal metastasis might be comparatively less pronounced and less likely to be associated with patient survival. As supportive evidence, the TMB scores were consistently low between subgroup patients, regardless of their pathological nodal status.

**FIGURE 3:**
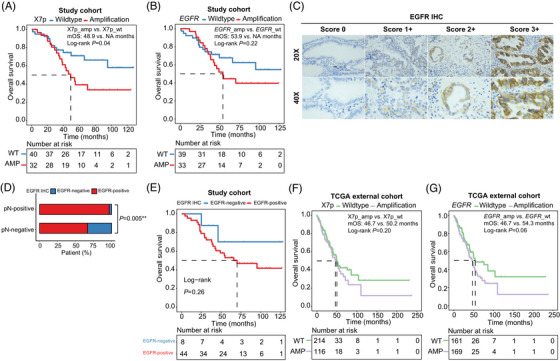
Chromosome 7p amplification enriched in pN‐positive patients was likely a metastatic driver in lung adenocarcinoma patients. (A, B) Kaplan‐Meier curve of overall survival in 72 lung adenocarcinoma patients (the study cohort) in strata of the presence of chromosome 7p (A) or epidermal growth factor receptor (*EGFR*) (B) amplification. (C) Immunohistochemistry (IHC) staining of formalin‐fixed, paraffin‐embedded (FFPE) samples showing negative, 1+, 2+, and 3+ membrane labelling for EGFR protein in lung adenocarcinoma. (D) The bar plot shows the proportion of patients with positive or negative EGFR expression based on IHC analyses in strata of pathological lymph node metastasis (pN) status. (E) Kaplan‐Meier curve of overall survival of 52 patients in strata of EGFR expression assessed by IHC. (F, G) Kaplan‐Meier curve of overall survival in 330 patients (the TCGA external cohort) in strata of the presence of chromosome 7p (C) or *EGFR* (D) amplification.

To further establish the relationship between the overexpression of EGFR, nodal metastasis, and inferior overall survival of patients, we performed immunohistochemistry analyses for EGFR on formalin‐fixed, paraffin‐embedded blocks obtained from 52 LUAD patients, including 31 pN‐positive and 21 pN‐negative patients (Figure 3C; Supporting Information). Notably, a statistically significant association was observed between positive pN status and EGFR stains (*p *= .005) (Figure 3D). Furthermore, there was a discernible trend among EGFR‐positive patients towards a shorter OS in comparison to those exhibiting negative staining; however, this difference was not statistically significant, most likely because of the small sample size (Figure 3E). Our findings align with those of Suzuki et al., who also reported that *EGFR* amplification and EGFR protein overexpression were associated with lymph node metastasis and shorter survival in patients with LUAD.^9^

We also employed the Cancer Genome Atlas‐Lung Adenocarcinoma dataset (hereafter referred to as The Cancer Genome Atlas [TCGA] dataset) to assess the applicability of the findings identified in our study cohort with extreme characteristics to real‐world clinical settings. Notably, 82.5% (99/120) of patients in the pN‐positive group had T1‐2 tumours, compared to only 21 patients with T3‐4, which was consistent with the argument that lymph node metastasis may not inherently correlate with larger tumours (Table S3). Consistently, patients with nodal metastasis exhibited considerably shorter OS than those without nodal involvement (32.7 vs. 76.2 months, *p *< .001) (Figure S3A). Regarding the genomic attributes assessed in the study cohort, X7p, and *EGFR* amplifications were also more commonly detected in pN‐positive patients and were associated with an unfavourable OS within the TCGA dataset (Figure S3B; Figure 3F,G). Consistent with findings within the study cohort, there exhibited little differences in TMB or CIS comparing both subgroups (Figure S3C,D). *MLXIPL* (MLX interacting protein‐like) amplifications, which were found to be more enriched in pN‐positive patients in the study cohort, showed a higher abundance in pN‐positive patients in the TCGA external dataset (Figure S3B). However, despite *MLXIPL* amplifications being likely associated with a favourable prognosis, no discernible disparity in OS between *MLXIPL*‐amplified and *MLXIPL*‐wildtype patients was noted in the TCGA cohort (Figure S3E,F).

Taken together, we present evidence indicating that gains on 7p11.2, particularly *EGFR* amplification, might serve as a potential metastatic driver in LUAD patients. It has been previously reported that chromosome 7p gains at 7p12‐p21, encompassing *EGFR*, are associated with lymph node metastasis in NSCLC patients.^10^ Worth noting the seemingly inconsistent results of chromosomal segments associated with lymph node metastasis might be attributed to the evolution of nomenclature for high‐resolution chromosomal banding and the methodological differences between WES and comprehensive genomic hybridization (CGH) in the assessment of CNVs. Indeed, CGH generally demands a relatively higher quantity and quality of DNA and tends to exhibit lower specificity compared to NGS. Nonetheless, both our study and Ubagai et al.^10^ reported a correlation between *EGFR* amplification and nodal metastasis. Given the feasibility of clinical samples and the cost‐effectiveness of NGS‐based methodologies, findings from our comprehensive WES analysis may provide additional clinical values if applied to biopsy specimens (i.e. tumour biopsies, plasma and pleural effusion) and assessed by targeted gene panels.

There are limitations to this study. First, the inherent constraints of WES being not able to identify non‐coding and structural variants necessitates the need to employ other molecular testing approaches, such as whole‐genome sequencing and combining targeted sequencing with WES for more unbiased results. Secondly, given the constraints posed by the small sample size of our study, it is imperative to undertake additional validation utilizing a larger patient cohort diagnosed with LUAD with or without nodal involvement. Thirdly, we acknowledge the discrepancies in the clinical characteristics of patients between the study cohort and the TCGA dataset. These differences may potentially lead to missed detection of enriched genetic alterations. However, clinical/molecular features that were highlighted in both cohorts despite these cohort differences are likely to be truly involved in lymph node metastasis.

## 1 CONCLUSION

To summarize, our findings suggest that pN status in LUAD patients may be a better predictor of survival outcomes than tumour size. Chromosome 7p CNVs, particularly *EGFR* amplifications, may be crucial in promoting the migration of primary lung tumours to nearby lymph nodes and are closely associated with the patient's overall survival. These findings highlight the importance of integrating clinical and genomic data into traditional radiologic modalities in the accurate diagnosis of pN status, which further benefits the clinical diagnosis, prognostic evaluation, and treatment decision‐making for LUAD patients.

## AUTHOR CONTRIBUTIONS

SJW and ZWZ designed this study. SJW, ZWZ, ZMM, DCZ, LW, YNL, YYM, and JZZ acquired clinical data and performed patient follow‐ups. SJW, ZWZ, SW, and RYG performed data analysis. All authors participated in data interpretation. SJW, ZWZ, SW, and QXO edited the manuscript. JFC conceived and supervised the study. All authors read and approved the final manuscript.

## CONFLICT OF INTEREST STATEMENT

Song Wang, Rongyun Guo, Sha Wang, Yedan Chen and Qiuxiang Ou are employees of Nanjing Geneseeq Technology Inc. The remaining authors declare no conflict of interest.

## FUNDING INFORMATION

This study was supported by the National Natural Science Foundation of China (No. NSFC81773144) and Capital Health Research and Development of Special Funds (2018‐2‐2155) to Jinfeng Chen. The funding agencies had no role in the study design, data collection, analyses, data interpretation, or decision to submit results.

## ETHICS STATEMENT

The study was approved by the Ethical Committee of Peking University Cancer Hospital & Institute (No. 2017KT78). All patients provided written informed consent to participate and publication.

## Supporting information

Supporting Information

## Data Availability

The datasets generated and/or analyzed during this current study are available from the corresponding author upon reasonable request.
