# Supplementary material for: Pathologic nodal metastasis assessment using tumour‐derived molecular features in patients with lung adenocarcinoma
Source: Clin Transl Med. 2024 Apr 1;14(4):e1638. doi: 10.1002/ctm2.1638 (PMC10983018; doi:10.1002/ctm2.1638)
Supplement: Supplementary file 1 — Supporting Information [file CTM2-14-e1638-s001.docx]

**Supplementary Material**

## **Supplementary Figures**

**
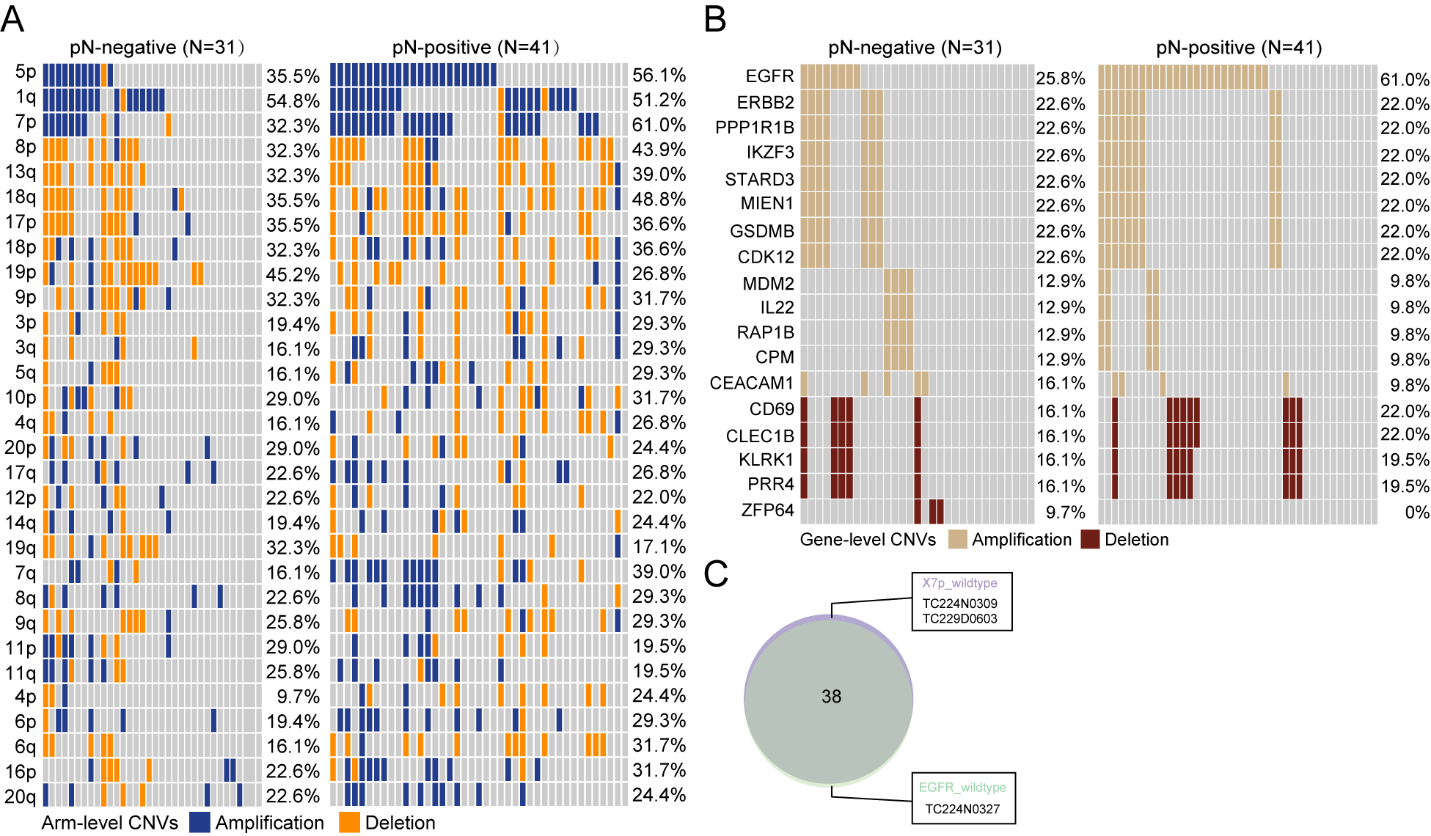
**

**Figure S1. Heatmap of copy-number changes in the study cohort**

**(A)** The heatmap shows the arm-level copy-number changes in baseline tumor samples from each patient, categorized based on their pathologic lymph node metastasis (pN) status. p, shorter arm region; q, longer arm region. **(B)** The heatmap shows the focal gene-level copy number variants (CNVs) of each patient identified by GISTIC analysis. **(C)** The Venn diagram shows the shared and unique patients in the study cohort without chromosome 7p or *EGFR* amplifications.

**
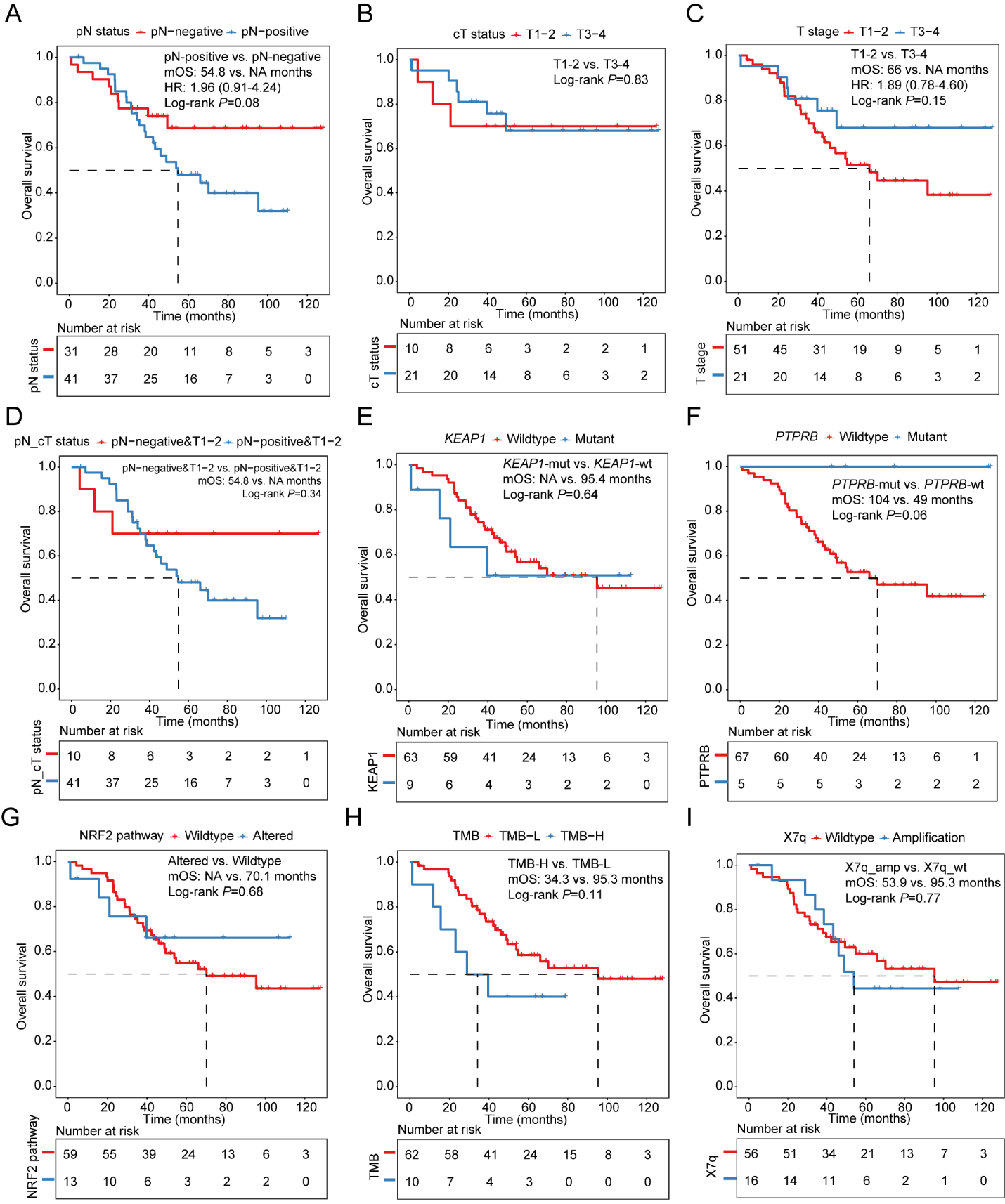
**

**Figure S2. Evaluating the association between clinical/molecular features and prognosis**

**(A)** Kaplan-Meier curve of overall survival in 72 lung adenocarcinoma (LUAD) patients (study cohort) in strata of pathologic lymph node metastasis (pN) status. **(B)** Kaplan-Meier curve of overall survival in pN-negative patients in strata of clinical T (cT) status. **(C)** Kaplan-Meier curve of overall survival in the study cohort in strata of T stage status. **(D)** Kaplan-Meier curve of overall survival of 51 LUAD patients with T1-2 disease in strata of pN status. **(E-I)** Kaplan-Meier curves of overall survival of the study cohort in strata of the presence of specific molecular features.

**
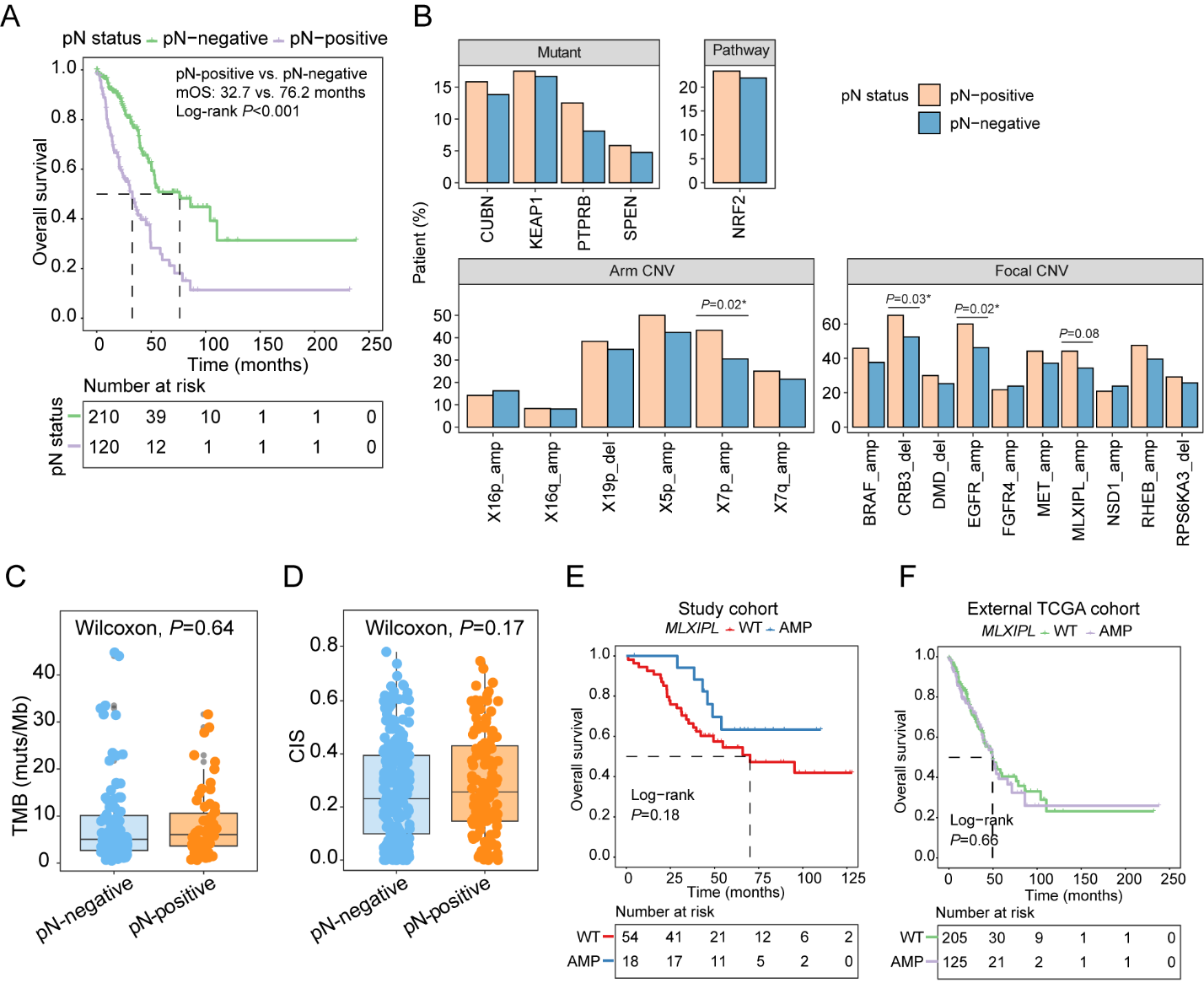
**

**Figure S3. External validation using the TCGA dataset**

**(A)** Kaplan-Meier curve of overall survival in 330 lung adenocarcinoma (LUAD) patients from the TCGA dataset in strata of pathologic lymph node metastasis (pN) status. **(B)** Enrichment analysis of selective genes, oncogenic signaling pathways, arm-level- and focal gene-level copy number variants (CNVs) in the TCGA external dataset between subgroup patients categorized based on their pN status. **(C)** The box plot of tumor mutation burden (TMB) in two patient subgroups. **(D)** The box plot of chromosomal instability score (CIS) in two patient subgroups. **(E, F)** Kaplan-Meier curve of overall survival in the study cohort (N=72) and in the external dataset (N=330) in strata of the presence of *MLXIPL* amplifications.

| **Table S1. *KEAP1* mutations identified in the study cohort** | | | | | | | |
| --- | --- | --- | --- | --- | --- | --- | --- |
| pN status | Start position | End position | Reference allele | Variant allele | AA Change | Mutation type | OncoKB |
| pN-negative | 10610321 | 10610321 | G | A | p. P130L | Missense | ~ |
| pN-negative | 10610163 | 10610163 | T | A | p. N183Y | Missense | ~ |
| pN-negative | 10600457 | 10600457 | A | - | p. V467Sfs*33 | Frameshift deletion | likely oncogenic |
| pN-negative | 10600471 | 10600471 | C | A | p. G462W | Missense | ~ |
| pN-negative | 10602446 | 10602446 | C | A | p. G378C | Missense | ~ |
| pN-negative | 10602755 | 10602763 | AGTGGCAGC | - | p.R272_S275delinsP | Inframe deletion | ~ |
| pN-negative | 10610227 | 10610227 | C | T | p.M161I | Missense | ~ |
| pN-positive | 10602773 | 10602773 | G | - | p. R269Gfs*8 | Frameshift deletion | likely oncogenic |
| pN-positive | 10610264 | 10610264 | T | C | p. E149G | Missense | ~ |
| pN, pathologic lymph node metastasis; AA, amino acid | | | | | | | |

## **Supplementary Tables**

| **Table S2. Univariate analysis of overall survival in features enriched in the study cohort** | | | | |
| --- | --- | --- | --- | --- |
| Feature | Type | Enriched population | HR (95% CI) for OS | *P*-value |
| TMB-high* | Clinical | pN-negative | 2.1 (0.84-5) | 0.11 |
| KEAP1* | Mutation | pN-negative | 1.3 (0.45-3.7) | 0.64 |
| SPEN | Mutation | pN-negative | 0.73 (0.17-3.1) | 0.66 |
| CUBN | Mutation | pN-negative | 1.1 (0.25-4.4) | 0.94 |
| PTPRB | Mutation | pN-negative | 0.00 (0.00-Inf) | >0.99 |
| NRF2_path* | Pathway | pN-negative | 0.8 (0.28-2.3) | 0.68 |
| X19p_del | Arm CNV | pN-negative | 0.72 (0.32-1.6) | 0.42 |
| DMD_del* | Arm CNV | pN-negative | 0.53 (0.26-1.1) | 0.08 |
| RPS6KA3_del | Focal CNV | pN-negative | 0.65 (0.32-1.3) | 0.23 |
| CRB3_del* | Focal CNV | pN-negative | 0.83 (0.39-1.8) | 0.65 |
| X7p_amp* | Arm CNV | pN-positive | 2.1 (1-4.2) | **0.04** |
| X16p_amp | Arm CNV | pN-positive | 1.6 (0.69-3.7) | 0.27 |
| X5p_amp | Arm CNV | pN-positive | 1.4 (0.71-2.8) | 0.33 |
| X7q_amp* | Arm CNV | pN-positive | 1.1 (0.51-2.5) | 0.77 |
| X16q_amp | Arm CNV | pN-positive | 2 (0.7-5.9) | 0.19 |
| MLXIPL_amp* | Focal CNV | pN-positive | 0.55 (0.22-1.3) | 0.18 |
| EGFR_amp* | Focal CNV | pN-positive | 1.5 (0.77-3.1) | 0.22 |
| FGFR4_amp* | Focal CNV | pN-positive | 1.3 (0.57-3.1) | 0.51 |
| NSD1_amp* | Focal CNV | pN-positive | 1.3 (0.57-3.1) | 0.51 |
| RHEB_amp* | Focal CNV | pN-positive | 1.2 (0.58-2.5) | 0.62 |
| MET_amp* | Focal CNV | pN-positive | 0.85 (0.38-1.9) | 0.70 |
| BRAF_amp* | Focal CNV | pN-positive | 1.1 (0.5-2.2) | 0.89 |
| *Significant genomic alterations in the enrichment analysis  Bold represents significant *P*-values in the Cox Proportional-Hazards model.  TMB, tumor mutation burden; X, chromosome; amp, amplification; del, deletion | | | | |

| **Table S3. Clinical characteristics of patients in the TCGA external dataset (N=330)** | | | | |
| --- | --- | --- | --- | --- |
|  | All (N=330) | pN-negative (N=210) | pN-positive (N=120) | *P*-value |
| Sex |  |  |  | 0.36 |
| Female | 166 (50.3%) | 110 (52.4%) | 56 (46.7%) | |
| Male | 164 (49.7%) | 100 (47.6%) | 64 (53.3%) | |
| Age at diagnosis, years |  |  |  | 0.80 |
| median (range) | 66 (33-87) | 67 (33-86) | 65 (42-87) |  |
| <60 | 93 (28.2%) | 58 (27.6%) | 35 (29.2%) | |
| ≥60 | 227 (68.8%) | 145 (69.1%) | 82 (68.3%) | |
| Unknown | 10 (3.0%) | 7 (3.3%) | 3 (2.5%) | |
| Clinical T stage |  |  |  | 0.04 |
| T1-2 | 289 (87.6%) | 190 (90.5%) | 99 (82.5%) | |
| T3-4 | 41 (12.4%) | 20 (9.52%) | 21 (17.5%) | |
| Clinical stage |  |  |  | <0.001 |
| I-II | 269 (81.5%) | 205 (97.6%) | 64 (53.3%) | |
| III | 61 (18.5%) | 5 (2.38%) | 56 (46.7%) | |
| Tumor site |  |  |  | 0.13 |
| LLL | 55 (16.7%) | 31 (14.8%) | 24 (20.0%) | |
| LUL | 77 (23.3%) | 45 (21.4%) | 32 (26.7%) | |
| RLL | 57 (17.3%) | 34 (16.2%) | 23 (19.2%) | |
| RML | 11 (3.3%) | 7 (3.3%) | 4 (3.3%) | |
| RUL | 122 (37.0%) | 89 (42.4%) | 33 (27.5%) | |
| Others | 2 (0.6%) | 2 (0.95%) | 0 (0.00%) | |
| Unknown | 6 (1.8%) | 2 (0.95%) | 4 (3.3%) | |
| LNM, lymph node metastasis; LLL, left lower lobe; LUL, left upper lobe; RLL, right lower lobe; RML, right middle lobe; RUL, right upper lobe | | | | |

## **Material and Methods**

### Patients and sample collection

Between 2013 and 2015, there were 72 untreated patients diagnosed with lung adenocarcinoma (LUAD) who underwent R0 resection for curative-intent treatment at the Peking University Cancer Hospital & Institute. Primary tumor tissues were collected from each patient and subjected to whole-exome sequencing (WES) analysis. The Cancer Genome Atlas-Lung Adenocarcinoma (TCGA-LUAD) dataset [1] consisting of 330 LUAD patients was used as an external dataset in this study. Overall survival (OS) was defined as the time from initial diagnosis until death, regardless of disease recurrence. Censored data were included for patients who had not progressed or died by the end of the study period. This study was conducted in accordance with the declaration of Helsinki and was approved by the Ethical Committee of Peking University Cancer Hospital & Institute (No. 2017KT78). Written consent form was obtained from each patient before sample collection.

### Library construction and whole-exome sequencing

DNA extraction, library preparation, and WES were performed following protocols as previously described [2]. Briefly, genomic DNA was extracted from formalin-fixed and paraffin-embedded (FFPE) sections and biopsy samples, with white blood cell samples as control, using the QIAamp DNA FFPE Tissue Kit (Qiagen Cat. No. 56404) and DNeasy Blood and Tissue Kit (Qiagen Cat. No. 69504) according to the manufacturer’s instructions. Genomic DNA was quantified by Qubit 3.0 using the dsDNA HS assay kit on a Qubit 3.0 fluorometer (Life Technology, US), followed by NGS library construction using the KAPA Hyper Prep kit (KAPA Biosystems). Exome enrichment capture was performed using the xGen Exome Research Panel v2 and Hybridization and Wash Reagents Kit (Integrated DNA Technologies) according to manufacturer’s protocol. Library fragment size was analyzed on a Bioanalyzer 2100 using the High Sensitivity DNA Kit (Agilent Technologies, Santa Clara, CA, 5067-4626). Sequencing was performed to the enriched libraries on the HiSeq4000 NGS platform (Illumina). The average coverage depth was 320X for tumors and 145X for normal blood controls.

### Mutation calling

FASTQ file quality control was performed using Trimmomatic [3]. Reads with a quality reading below 20 and/or N bases were removed. Paired-end reads were then aligned to the reference human genome (build hg19) using the Burrows-Wheeler Aligner (BWA, <https://github.com/lh3/bwa/tree/master/bwakit>) with default parameters. Picard (https://broadinstitute.github.io/picard/) was used to remove PCR duplications, and local alignment around indels and base quality score recalibration was performed using GATK3 (https://software.broadinstitute.org/gatk/). Somatic single nucleotide variant (SNV) and insertion/deletions calling were performed using VarScan2 and Mutect2 [4]. Common SNVs present in >1% population frequency in the 1,000 Genomes Project [5] or the Exome Aggregation Consortium (ExAC) 65,000 exomes database were excluded. The curated SNV list underwent additional refinement by specifically focusing on somatic alterations within ten canonical signaling pathways that signify common cancer hallmarks [6]. The final compilation of SNVs went through a manual review process using the Integrative Genomics Viewer. Tumor mutation burden (TMB) was calculated as the total number of nonsynonymous mutations divided by the length of the genomic target region.

### Copy number alteration analysis

Copy number variation (CNV) analysis was performed using FACETS [7]. For a given chromosome, a CNV event was classified as “chromosomal” if at least 60% of its segments had a consistent degree of copy number alteration. For focal-level CNVs, amplification or deletion was characterized based on the total copy number (tcn) and ploidy calculated by the FACETS algorithm as previously described [8]. In brief, amplification and deletion were identified if the value of (tcn-ploidy) was ≥1 or ≤-1, respectively. Significantly amplified or deleted CNVs were identified using the Genome Identification of Significant Targets in Cancer (GISTIC) 2.0 algorithm [9] with parameters as follows: q-value < 0.25, ta = 0.25, td = 0.25, cap = 6, broad = 1, brlen = 0.7, and genegistic = 1. The genes identified within GISTIC 2.0 peaks were subjected to further investigation if included in the oncogenic signaling pathways [6]. The chromosomal instability score (CIS) was calculated using CNV events, which was defined as the proportion of the length of the genome with segmented copy number alterations.

### Immunohistochemistry

Immunohistochemistry (IHC) analyses for epidermal growth factor receptor (EGFR) were performed on FFPE samples obtained from 52 patients with LUAD (31 pN-positive and 21 pN-negative) using anti-EGFR monoclonal antibody EP38Y (Abcam # ab52894) according to the manufacturer’s instructions. As previously described [10, 11], EGFR staining was scored independently by three pathologists in a blinded fashion based on membranous staining intensity per the following criteria: 0 = complete absence of staining or faint staining in <10% of tumor cells; 1+ = faint, partial membranous staining in >10% of tumor cells; 2+ = weak, complete membranous staining in >10% of tumor cells; 3+ = intense, complete membranous staining in >10% of tumor cells. Tumors with 0 scores were interpreted as negative expression, and tumors with 1+, 2+, and 3+ were interpreted as positive expression.

### Statistical analysis

All statistical analyses were performed in R (version 4.1.3). Fisher’s exact tests were used to compare the frequencies of categorical measures among different groups, and Wilcoxon rank sum tests were used to compare the distribution of continuous data. Kaplan-Meier curves were used to analyze the OS of different patient groups, and the statistical difference was assessed using the log-rank test. A two-sided *P* value of less than 0.05 was considered significant for all tests unless indicated otherwise (**P*<0.05, ***P*<0.01, ****P*<0.001).

## **References**

1. K. Tomczak, P. Czerwinska and M. Wiznerowicz, *The Cancer Genome Atlas (TCGA): an immeasurable source of knowledge.* Contemp Oncol (Pozn), 2015. **19**(1A): p. A68-77.

2. Z. Yang, N. Yang, Q. Ou, Y. Xiang, T. Jiang, X. Wu, et al., *Investigating Novel Resistance Mechanisms to Third-Generation EGFR Tyrosine Kinase Inhibitor Osimertinib in Non-Small Cell Lung Cancer Patients.* Clin Cancer Res, 2018. **24**(13): p. 3097-3107.

3. A.M. Bolger, M. Lohse and B. Usadel, *Trimmomatic: a flexible trimmer for Illumina sequence data.* Bioinformatics, 2014. **30**(15): p. 2114-20.

4. K. Cibulskis, M.S. Lawrence, S.L. Carter, A. Sivachenko, D. Jaffe, C. Sougnez, et al., *Sensitive detection of somatic point mutations in impure and heterogeneous cancer samples.* Nat Biotechnol, 2013. **31**(3): p. 213-9.

5. C. Genomes Project, A. Auton, L.D. Brooks, R.M. Durbin, E.P. Garrison, H.M. Kang, et al., *A global reference for human genetic variation.* Nature, 2015. **526**(7571): p. 68-74.

6. F. Sanchez-Vega, M. Mina, J. Armenia, W.K. Chatila, A. Luna, K.C. La, et al., *Oncogenic Signaling Pathways in The Cancer Genome Atlas.* Cell, 2018. **173**(2): p. 321-337 e10.

7. R. Shen and V.E. Seshan, *FACETS: allele-specific copy number and clonal heterogeneity analysis tool for high-throughput DNA sequencing.* Nucleic Acids Res, 2016. **44**(16): p. e131.

8. W.F. Tang, M. Wu, H. Bao, Y. Xu, J.S. Lin, Y. Liang, et al., *Timing and Origins of Local and Distant Metastases in Lung Cancer.* J Thorac Oncol, 2021. **16**(7): p. 1136-1148.

9. C.H. Mermel, S.E. Schumacher, B. Hill, M.L. Meyerson, R. Beroukhim and G. Getz, *GISTIC2.0 facilitates sensitive and confident localization of the targets of focal somatic copy-number alteration in human cancers.* Genome Biol, 2011. **12**(4): p. R41.

10. J. Yu, S. Kane, J. Wu, E. Benedettini, D. Li, C. Reeves, et al., *Mutation-specific antibodies for the detection of EGFR mutations in non-small-cell lung cancer.* Clin Cancer Res, 2009. **15**(9): p. 3023-8.

11. M. Brevet, M. Arcila and M. Ladanyi, *Assessment of EGFR mutation status in lung adenocarcinoma by immunohistochemistry using antibodies specific to the two major forms of mutant EGFR.* J Mol Diagn, 2010. **12**(2): p. 169-76.
